# Runx1 is required for progression of CD41^+^ embryonic precursors into HSCs but not prior to this

**DOI:** 10.1242/dev.110841

**Published:** 2014-09

**Authors:** Anna Liakhovitskaia, Stanislav Rybtsov, Tom Smith, Antoniana Batsivari, Natalia Rybtsova, Christina Rode, Marella de Bruijn, Frank Buchholz, Sabrina Gordon-Keylock, Suling Zhao, Alexander Medvinsky

**Affiliations:** 1 MRC Centre for Regenerative Medicine, University of Edinburgh, Edinburgh EH16 4UU, UK; 2 MRC Molecular Haematology Unit, Weatherall Institute of Molecular Medicine, University of Oxford, Oxford OX3 9DS, UK; 3 Max Planck Institute of Molecular Cell Biology and Genetics, 01307 Dresden, Germany

**Keywords:** AGM region, CD41, HSC, Runx1, Mouse

## Abstract

Haematopoiesis in adult animals is maintained by haematopoietic stem cells (HSCs), which self-renew and can give rise to all blood cell lineages. The AGM region is an important intra-embryonic site of HSC development and a wealth of evidence indicates that HSCs emerge from the endothelium of the embryonic dorsal aorta and extra-embryonic large arteries. This, however, is a stepwise process that occurs through sequential upregulation of CD41 and CD45 followed by emergence of fully functional definitive HSCs. Although largely dispensable at later stages, the Runx1 transcription factor is crucially important during developmental maturation of HSCs; however, exact points of crucial involvement of Runx1 in this multi-step developmental maturation process remain unclear. Here, we have investigated requirements for Runx1 using a conditional reversible knockout strategy. We report that Runx1 deficiency does not preclude formation of VE-cad+CD45−CD41+ cells, which are phenotypically equivalent to precursors of definitive HSCs (pre-HSC Type I) but blocks transition to the subsequent CD45+ stage (pre-HSC Type II). These data emphasise that developmental progression of HSCs during a very short period of time is regulated by precise stage-specific molecular mechanisms.

## INTRODUCTION

Embryonic development of the haematopoietic stem cell lineage occurs through sequential maturation stages (Cumano and Godin, 2007; Dzierzak and Speck, 2008; Medvinsky et al., 2011). By mid-gestation, definitive HSCs (dHSCs) emerge in the aorta-gonad-mesonephros (AGM) region, as well as in placenta, large extra-embryonic vessels, yolk sac and perhaps head (de Bruijn et al., 2000; Dzierzak and Robin, 2010; Gekas et al., 2005; Gordon-Keylock et al., 2013; Li et al., 2012; Medvinsky and Dzierzak, 1996). The current prevailing view that HSCs originate in the dorsal aorta is supported by strong evidence in lower vertebrates, mouse and human (Bertrand et al., 2010, 2005; Ciau-Uitz et al., 2000; Dieterlen-Lievre, 1975; Ivanovs et al., 2011; Kissa and Herbomel, 2010; Medvinsky and Dzierzak, 1996; Medvinsky et al., 1993; Swiers et al., 2013; Taoudi and Medvinsky, 2007). Definitive HSCs (dHSC) originate from the mesoderm that generates the VE-cadherin+ endothelium, part of which becomes haematogenic and in turn generates the haematopoietic compartment marked by CD41 and subsequently by CD45 (Cumano and Godin, 2007; Dzierzak and Speck, 2008; Medvinsky et al., 2011; Mikkola et al., 2003; Rybtsov et al., 2011; Taoudi et al., 2008). Runx1 is a transcription factor playing a key role in development of the haematopoietic system; however, it is largely dispensable for the maintenance of adult bone marrow HSCs (Ichikawa et al., 2004; North et al., 1999; Okuda et al., 1996; Putz et al., 2006; Wang et al., 1996). Germline *Runx1* homozygous deletion blocks both erythro-myeloid haematopoietic progenitors (CFU-C) and HSC formation (Cai et al., 2000), which leads to severe anaemia and embryonic death by E12.5. Conditional genetic and cell fate analysis using VE-cadherin-Cre deletor mice indicates that Runx1 is crucial for the endothelial-haematopoietic transition during HSC formation (Chen et al., 2009). However, this transition involves at least three sequential stages of maturation marked by continuous expression of VE-cadherin and sequential upregulation of haematopoietic markers, first CD41 (pre-HSC Type I: VE-cad+CD41+CD45−) and subsequently CD45 (pre-HSC Type II: VE-cad+CD45+), before they become fully functional definitive HSCs (Rybtsov et al., 2011; Taoudi et al., 2008). Time specific-induced inactivation shows that Runx1 is critically important for HSC development even at E11.5 (Tober et al., 2013). This raises the question of exactly when HSC development in *Runx1* null embryos is blocked. Here, using Runx1 conditional reversible knockouts (Liakhovitskaia et al., 2009; Samokhvalov et al., 2006), we show that contrary to previously held opinion, the HSC lineage in *Runx1* knockout embryos develops up to the point when it expresses CD41, considered to be a haematopoietic commitment marker in development (Ferkowicz et al., 2003; Mikkola et al., 2003; Mitjavila-Garcia et al., 2002). Although the CD41+ cell population is smaller in knockout embryos, it is clearly detectable and is less apoptotic than haematopoietic cells in wild-type embryos. Accordingly, conditional restoration of the *Runx1* locus using CD41-Cre deletor mice rescues definitive HSCs (for experimental design, see supplementary material Fig. S1A). In summary, we show that in the complete absence of Runx1, haematopoietic specification of the HSC lineage in the embryo is initiated towards the CD41+ stage, but cannot progress.

## RESULTS AND DISCUSSION

**Fig. 1.**
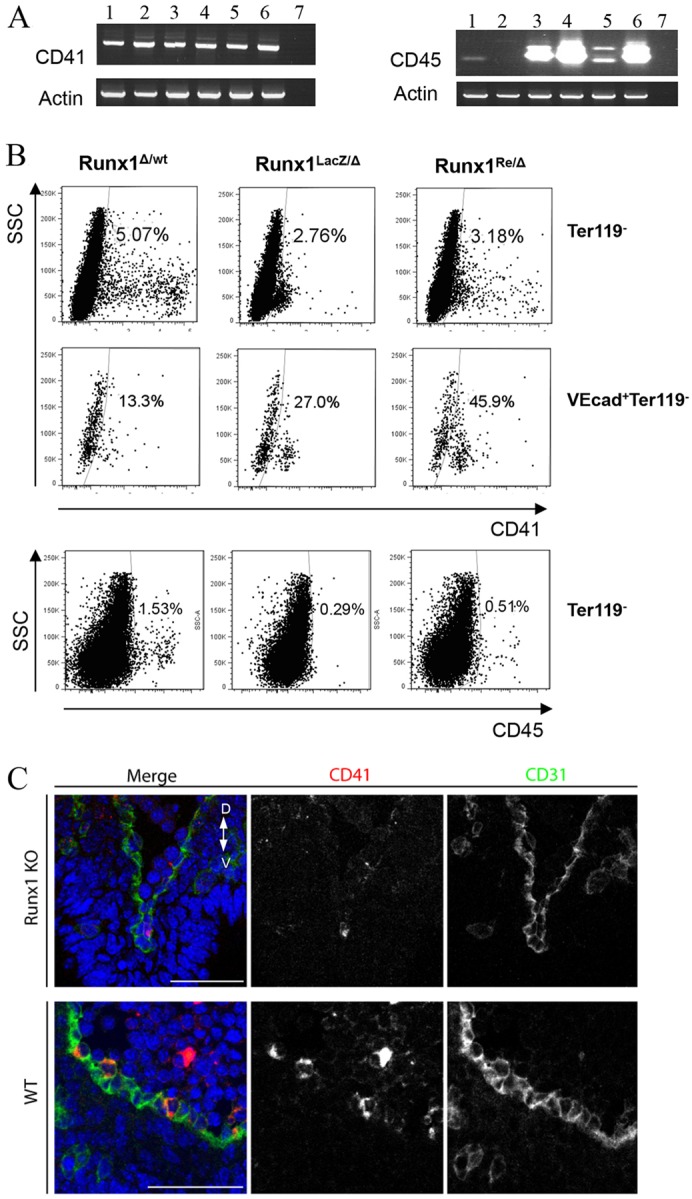
***Runx1* knockout embryos develop CD41^+^ cells.** (A) CD41 (left panel) and CD45 (right panel) mRNA are detected in wild-type and *Runx1* knockout embryos by RT-PCR. E9.5 *Runx1* knockout body and yolk sac (YS) (lanes 1 and 2, respectively); E9.5 wild-type body and YS (lanes 3 and 4, respectively); E11.5 *Runx1* knockout and wild-type YS (lanes 5 and 6, respectively); H_2_0 control (lane 7). (B) Flow cytometry analysis of *Runx1* heterozygous, *Runx1* knockout [Runx1^LacZ/Δ^] and rescued [CD41-Cre::Runx1^LacZ/Δ^] embryos (E10.5 AGM regions) obtained through crossing as outlined in supplementary material Fig. S1. (Top row) Runx1^wt/Δ^ and rescued Runx1^Re/Δ^ embryos contain both CD41^lo^ and CD41^hi^ cells; however, knockout Runx1^LacZ/Δ^ embryos develop mainly CD41^lo^ cells (7AAD+Ter119+ cells are excluded). (Middle row) Runx1^LacZ/Δ^ embryos contain VEcad^+^CD45^−^CD41^lo^ cells bearing the pre-HSCs Type I phenotype (7AAD+Ter119+VE-cad- cells are excluded). (Bottom row) CD45^+^ cells are absent in *Runx1* knockout embryos but are rescued in [CD41-Cre::Runx1l^LacZ/Δ^] embryos (7AAD+Ter119+ cells are excluded). (C) CD41^+^ and CD31^+^ cells in the E10.5 dorsal aorta of wild-type and Runx1^LacZ/Δ^ knockout embryos (confocal microscopy). Scale bars: 50 µm.

**Fig. 2.**
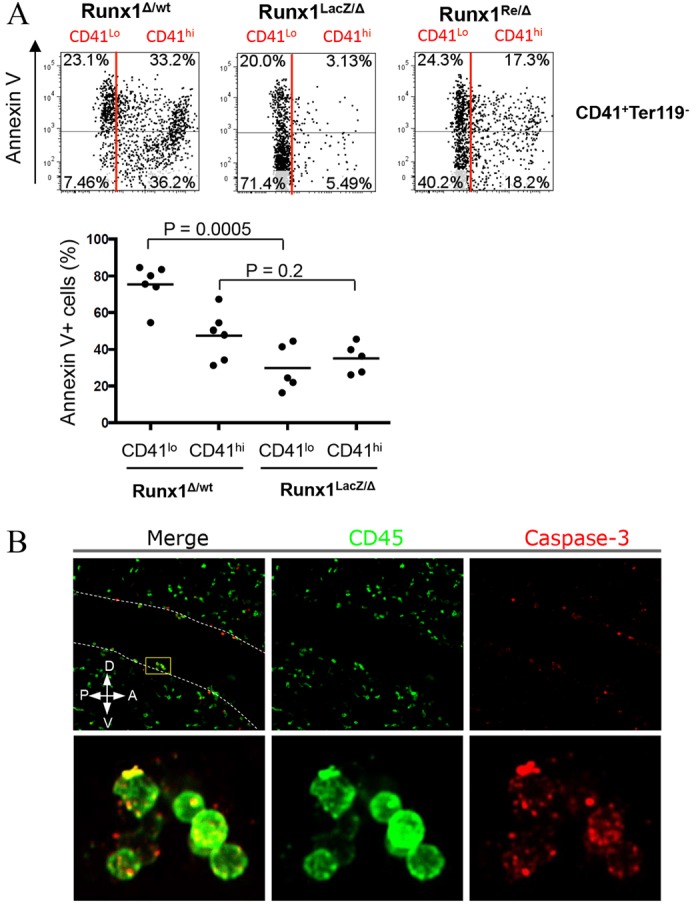
**Development of haematopoietic cells in *Runx1* knockout and rescued embryos.** (A) Representative plots of annexin V staining in CD41^+^Ter119^−^ cells in E10.5 AGM regions (7AAD+Ter119+CD41− cells were gated out). (Top) The CD41^lo^ population in Runx1^LacZ/Δ^ embryos contains a smaller proportion of annexin V^+^ cells than in heterozygous Runx1^Δ/wt^ embryos (red line separates CD41^lo^ and CD41^hi^ subsets); the same tendency but to a smaller degree is observed in the CD41^hi^ population. (Bottom) Proportion of annexin V^+^ cells in CD41^lo^ and CD41^hi^ fractions in knockout Runx1^LacZ/Δ^ and control Runx1^Δ/wt^ E10.5 AGM regions. Each circle represents an individual embryo. Data were obtained from five independent experiments. (B) Active caspase 3 expression in intra-aortic haematopoietic clusters in the wild-type E11.5 AGM region (confocal microscopy). Dotted lines show the endothelial lining of the dorsal aorta. D-V and A-P indicate the dorsoventral and anterioposterior axes, respectively.

We investigated whether Runx1-deficient embryos show any haematopoietic commitment beyond primitive erythropoiesis (Okuda et al., 1996) and detected by RT-PCR an early haematopoietic marker, CD41, in E11.5 *Runx1* knockout embryos (Fig. 1A). Flow cytometry analysis confirmed the presence of a low CD41-expressing (CD41^lo^) cell population and very few bright CD41-expressing (CD41^hi^) cells in *Runx1* knockout embryos compared with heterozygous littermate controls (Fig. 1B; supplementary material Fig. S2A). CD41^lo^ cells in *Runx1* knockout embryos could be identified in the area of the dorsal aorta using immunofluorescence (Fig. 1C). By contrast, the CD45^+^ population is practically non-detectable (Fig. 1A,B). We found that in *Runx1* heterozygous and wild-type embryos, a large proportion of both CD41+ and CD45+ haematopoietic cells were apoptotic, as evidenced by annexin V staining (Fig. 2A; supplementary material Fig. S2A; data not shown) and active caspase 3 staining of many cells in intra-aortic clusters (Fig. 2B). In individual embryos, 25-55% of intra-aortic clusters contained at least one active caspase 3+ cell, and some clusters were entirely apoptotic (supplementary material Fig. S2B). In layers surrounding the dorsal aorta, 31-47% of CD45+ cells were apoptotic (supplementary material Fig. S2B). However, the CD41^lo^ population in *Runx1* knockout embryos was less apoptotic than in littermate controls (Fig. 2A). A similar tendency was observed in CD41^hi^ cells, which were produced in considerably smaller numbers in *Runx1* knockout embryos (Fig. 2A). Flow cytometry analysis has shown that phenotypic equivalents of pre-HSC Type I (VE-cad^+^CD45^−^CD41^lo^) can be detected in *Runx1* mutants (Fig. 1B, middle panel). We therefore investigated whether the block in HSC development occurs in the CD41 compartment and can be overcome by restoration of Runx1 expression in CD41^+^ cells. To this end, [CD41-Cre :: Runx1^LacZ/Δ^] embryos were generated in which both *Runx1* alleles are non-functional, of which one is stably deleted (Runx1^Δ^) and the other (Runx1^LacZ^) can be reactivated through Cre-mediated recombination, hereafter referred to as Runx1^Re^ (see Materials and Methods; supplementary material Fig. S1) (Samokhvalov et al., 2006). In contrast to Runx1^LacZ/Δ^ knockout embryos, Runx1^Re/Δ^ embryos showed clear signs of rescued haematopoiesis. Both the E10.5 AGM region and the yolk sac developed CD41^hi^ cells similar to *Runx1* heterozygous littermates (Fig. 1B and data not shown). CD45^+^ populations were also observed in the AGM region and yolk sac of rescued embryos (Fig. 1B and data not shown). In contrast to *Runx1* mutants, Runx1^Re/Δ^ embryos were no longer dying by E12.5 and survived until birth, but as expected were not found alive after that due to other non-haematopoietic defects (Liakhovitskaia et al., 2010).

**Fig. 3.**
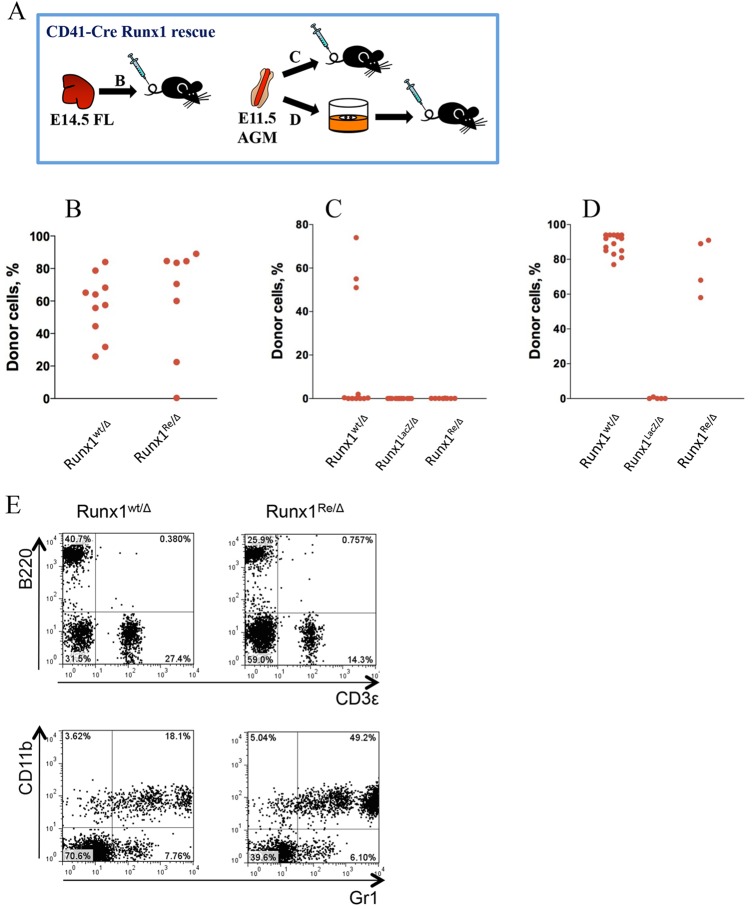
**HSCs are rescued in [CD41-Cre::Runx1^LacZ/Δ^] embryos.** (A) Experimental design: left, transplantation of E14.5 foetal livers; right, transplantation of fresh and cultured E11.5 AGM regions. (B-D) Long-term donor-derived haematopoietic repopulation with (B) E14.5 foetal livers from control Runx1^wt/Δ^ and rescued Runx1^Re/Δ^ embryos; (C) uncultured E11.5 AGM region cells; and (D) cultured E11.5 AGM region cells. The donor cell contribution (%) into the peripheral blood of recipient mice is shown (for details of culture, transplantation and analysis, see Materials and Methods). Each symbol represents one recipient mouse. Data obtained from three independent experiments. (E) Representative examples of long-term multilineage donor-derived haematopoietic repopulation ([CD41-Cre::Runx1^LacZ/Δ^] E14.5 foetal liver, 14 weeks post-transplantation). Gating was carried out on 7AAD-Ly5.2+ cells.

To test whether development of HSCs was rescued, foetal liver cells from E14.5 Runx1^Re/Δ^ embryos were transplanted into irradiated recipients. This led to successful long-term multi-lineage donor-derived engraftment, with only one exception (Fig. 3B). All donor-derived lymphoid and myeloid lineages were represented similar to control *Runx1* heterozygous transplants (Fig. 3E). Transplantations into secondary recipients also gave multi-lineage donor-derived haematopoietic engraftment (data not shown). However, when we tested whether HSCs are rescued in the AGM region, we found that, in contrast to *Runx1* heterozygous AGM regions, transplantation of E11.5 Runx1^Re/Δ^ AGM regions did not produce haematopoietic repopulation (Fig. 3C). One out of five yolk sacs and one of six placentas were able to repopulate irradiated recipients (not shown). To test the possibility of delayed HSC development in rescued embryos, AGM region explants were cultured for 4 days in conditions supporting HSC development followed by transplantation into irradiated recipients (Fig. 3D). All four recipients transplanted showed high levels of donor-derived multi-lineage haematopoietic engraftment, thus demonstrating the presence of rescued pre-HSCs in the AGM region of Runx1^Re/Δ^ embryos (Fig. 3D). None of the five Runx1^LacZ/Δ^ AGM explants, which did not harbour the Cre transgene, were able to repopulate recipient mice.

In previous reports, inactivation of Runx1 in the VE-cad+ population suggested that Runx1 is essential for endothelio-haematopoietic transition but not subsequently, when CFU-Cs and HSCs start expressing Vav (Chen et al., 2009). However, continuous expression of VE-cadherin over several HSC developmental stages within the VE-cad/Vav expression time window obscures the exact initial point at which Runx1 deficiency blocks this process. Our data demonstrate that, in *Runx1* knockout embryos, initial haematopoietic specification does occur. Indeed, while Runx1^LacZ/Δ^ knockout embryos develop VE-cad^+^CD41^low^CD45^−^ cells, low numbers of CD45+ cells are generated at E9.5 but disappear by E11.5. However, CD41^low^ cells in *Runx1* null embryos are stably present and are less apoptotic than in control *Runx1* heterozygous embryos. This explains why successful restoration of the *Runx1* functional allele in the CD41^+^ population of mutant embryos rescued both CFU-Cs and definitive HSCs. Therefore, *in vivo* Runx1 is required for transition of CD41^+^ cells into the CD45^+^ cells but not prior to that. This result contradicts previous reports indicating that Runx1 deficiency blocks transition from CD41-negative endothelial into CD41^+^ haematopoietic cells (Bertrand et al., 2008; Lancrin et al., 2009). This discrepancy could be due to the use of an ES cell system as a model system in which haematopoietic differentiation may deviate from the *in vivo* development, or due to differences in sensitivity of methods of CD41 detection. However, an early study reported that Runx1-deficient ES cells can generate CD41+ cells lacking Kit expression (Mikkola et al., 2003). Of note, some Runx1-deficient zebrafish do recover from a larval ‘bloodless’ phase and develop to fertile adults with multilineage haematopoiesis (Sood et al., 2010), which might be explained at least partly by initiation of the haematopoietic programme in the absence of Runx1. It would be interesting to investigate whether *Runx1*-deficient cells in zebrafish, which die attempting to undergo endothelial-haematopoietic transition in the dorsal aorta, acquire the CD41^+^ phenotype prior to that (Kissa and Herbomel, 2010). Apoptosis observed during normal early haematopoietic development is an interesting phenomenon. Significant reduction of apoptosis in haematopoietic cells of Runx1-deficient embryos concurrent with blockade of haematopoietic differentiation suggests that apoptosis is an attribute of haematopoietic differentiation and not of the most immature CD41 fraction.

In summary, we demonstrate that, in the absence of Runx1, the HSC lineage progresses to the CD41+ stage but ceases further development. Therefore, transition from the CD41− endothelium into the haematopoietically committed CD41+ stage is Runx1 independent. This study provides a better understanding of Runx1-dependent check-points during HSC development, which may be required for generating definitive HSCs from pluripotent ES/iPS cells *in vitro*.

## MATERIALS AND METHODS

### Mice

All mice used to generate embryos were bred to the C57BL/6 (CD45.2/2) background. Transgenic mice used in this study have been described previously: CD41-Cre deletor mice (Emambokus and Frampton, 2003; Rybtsov et al., 2011); activatable Runx1^LacZ/wt^ mice (Samokhvalov et al., 2006); and conditional Runx1^fl/fl^ knockout mice (Putz et al., 2006). Runx1^fl/wt^ mice were used to generate Runx1^Δ/wt^ by Cre-mediated excision. For experimental crossings, we always used [CD41-Cre :: Runx1^Δ/wt^] males and Runx1^LacZ/wt^ females (supplementary material Fig. S1). The morning of discovery of the vaginal plug was designated embryonic day 0.5. [CD41-Cre :: Runx1^Δ/LacZ^] embryos in the figures and figure legends are presented as Runx1^Re/Δ^ for brevity. Mice were bred and used in experiments under UK Home Office regulations with approval of the University of Edinburgh Ethical Review Committee.

### Long-term repopulation assay

Cell suspensions from embryos at different stages were injected into irradiated adult recipients (*CD45.1/1*) either directly (suspensions from AGM region or E14.5 foetal livers) or after culture (E11.5 AGM region explants), along with 80,000 *CD45.2/1* bone marrow carrier cells. Recipients were irradiated by a split dose (600+550 rad with 3 h interval) of γ irradiation. Donor-derived chimerism was monitored in blood at different time points after transplantation using LSRFortessa (BD). The peripheral blood was collected by bleeding the lateral tail vein into 500 µl of 5 mM EDTA/PBS, and erythrocytes were depleted using PharM Lyse (BD). Cells were stained with anti-CD16/32 (Fc-block), CD45.1-APC (clone A20) and anti-CD45.2-PE (clone 104) monoclonal antibodies (eBioscience). Appropriate isotype controls were used. Dead cells were excluded using 7AAD (eBioscience).

### Flow cytometry analysis

Donor-derived contribution into different hematopoietic lineages in blood or organs was determined by exclusion of recipient and carrier CD45.1^+^ cells and staining with lineage-specific monoclonal antibodies to Mac1, CD3e, Gr1, B220 and Ter119 conjugated with PE, FITC, APC or biotin. Biotinylated antibodies were detected by incubation with streptavidin APC or PE (BD). All analyses were performed using FlowJo software (Tree Star). Statistical analyses were performed in GraphPad Prism6 software.

### AGM region explant culture

E11.5 AGM regions were dissected and cultured for 5 days on floating 0.8 µm Millipore membranes at the liquid-gas interface with IMDM^+^ media consisting of 20% FCS, L-Gln, P/S IMDM and growth factors (100 ng/ml IL-3, 100 ng/ml SCF, and 100 ng/ml Flt3 ligand; all from PeproTech) as previously described (Taoudi et al., 2008). After culture, explants were dissociated enzymatically as previously described and long-term repopulation assays were peformed.

### Genotyping of embryos and assessment of Cre-mediated recombination

Genotyping was performed by Southern blotting as described previously (Liakhovitskaia et al., 2009). Specific recombination in [CD41-Cre :: Runx1^Δ/LacZ^] embryos was always controlled by analysing the recombination in blood and separately in the tail of the embryo.

### Confocal microscopy

Whole-mount immunostaining was performed as previously described (Yokomizo and Dzierzak, 2010). Briefly, embryos were dissected from the yolk sac, fixed with 2% paraformaldehyde (PFA), dehydrated in ascending concentration of methanol, and the head, limbs and one lateral body wall removed. Samples were then rehydrated by 50% methanol, washed with PBS and blocked in 50% FCS/0.5% Triton X-100. Embryos were incubated overnight with primary antibodies: unconjugated rabbit anti-mouse active caspase 3 (C92-605, BD Pharmingen, 1:100) and goat anti-mouse CD45 (AF114, R&D Systems, 1:100). Secondary antibodies used were anti-rabbit NL557 (NL004, R&D Systems, 1:100) and anti-rat Alexa 488 (A-21208, Invitrogen, 1:100). Then the embryos were washed, dehydrated in methanol and cleared with BABB solution. Images were acquired using an inverted confocal microscope (Leica SP8) and processed using Volocity software.

For immunostaining on sections, embryos were fixed in 4% PFA, washed with PBS, incubated in 15% sucrose, embedded in OCT compound and snap-frozen on dry-ice/ethanol. Frozen sections (10 µm) mounted on slides were washed in 10% FCS/PBS (with penicillin/streptomycin), incubated in PBS containing 10% FCS, penicillin/streptomycin and 0.05% Tween 20, and peroxidase quenched with 3% hydrogen peroxide to be used with Tyramide Amplification kit (Molecular Probes, #T30955). After applying blocking buffer and washing in PBS, slides were incubated with anti-mouse CD31 antibody (BD #553370, 1:30) for 1 h and washed with PBS. After incubation with secondary Alexa Fluor 488 goat anti-rat antibody for 1-2 h and washing with PBS, sections were blocked with PBS containing 10% FCS, 0.05% Tween 20 and 5% normal rat serum. Endogenous avidin and biotin were blocked for 30 min each (Abcam, #ab3387). Following 1 h staining with biotinylated CD41 antibody (eBioscience, #13-0411-82, 1:100), sections were incubated with SAV-HRP and Tyramide Alexa Fluor 555 according to the Tyramide Amplification kit instructions. Sections were mounted with HardSet Vectashield containing DAPI. Images were taken on Zeiss 510 confocal microscope using the 63× oil objective and processed with ZEN2011, Adobe Photoshop and Illustrator.

### RT-PCR analysis

RNA was islolated using the RNeasy mini kit (Qiagen) and treated with DNase I (Ambion). DNA-free RNA (1 μg) was used as a template for the random primed reverse transcription reaction using the Retroscript first-strand synthesis kit for RT-PCR (Ambion). Ten percent of the RT reaction were used for PCR with CD41 (5′-GTTTGGGAAGAAGGAAGATGGC-3′ and 5′-ATTTCCACCGCTCCCAAGG-3′), CD45 (5′-GGCAAACACC-TACACCCAGTGA-3′ and 5′-CCATGGGGTTTAGATGCAG-3′) and actin (5′-CCAGAGCAAGAGAGGTATC-3′ and 5′-TGGAAGGTGGAC-AGTGAG-3′) primers.

## Supplementary Material

Supplementary Material
