# Supplementary material for: Runx1 is required for progression of CD41+ embryonic precursors into HSCs but not prior to this
Source: Development. 2014 Sep;141(17):3319–23. doi: 10.1242/dev.110841 (PMC4199125; doi:10.1242/dev.110841)
Supplement: Supplementary Material [file supp_141_17_3319__index.html]

Runx1 is required for progression of CD41+ embryonic precursors into HSCs but not prior to this — Supplementary Material 

# Runx1 is required for progression of CD41+ embryonic precursors into HSCs but not prior to this

## DEV110841 Supplementary Material

**Files in this Data Supplement:**

- **Supplementary Material**
