# Supplementary material for: Runx1 is required for progression of CD41+ embryonic precursors into HSCs but not prior to this
Source: Development. 2014 Sep;141(17):3319–23. doi: 10.1242/dev.110841 (PMC4199125; doi:10.1242/dev.110841)
Supplement: Supplementary Material [file supp_141.17.3319_DEV110841.pdf]

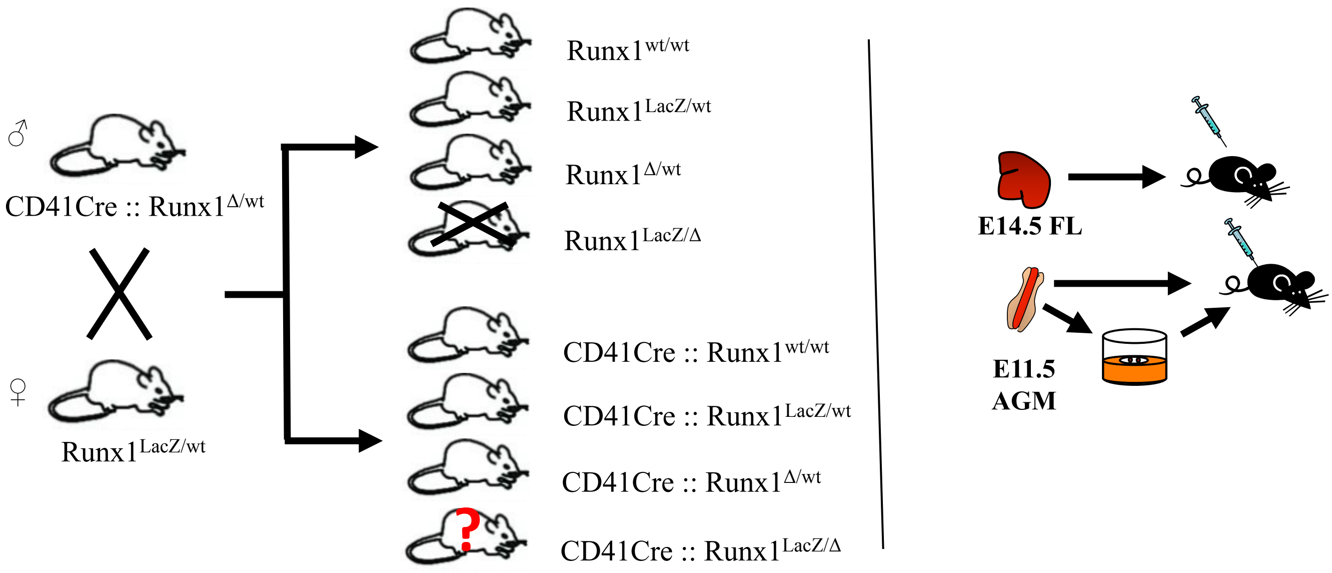

Fig. S1. Crossing strategy. Mouse crossing to reactivate Runx1^{LacZ} locus *in vivo* in the CD41 compartment. Statistically, every 1 of 8 embryos is reactivated.

A

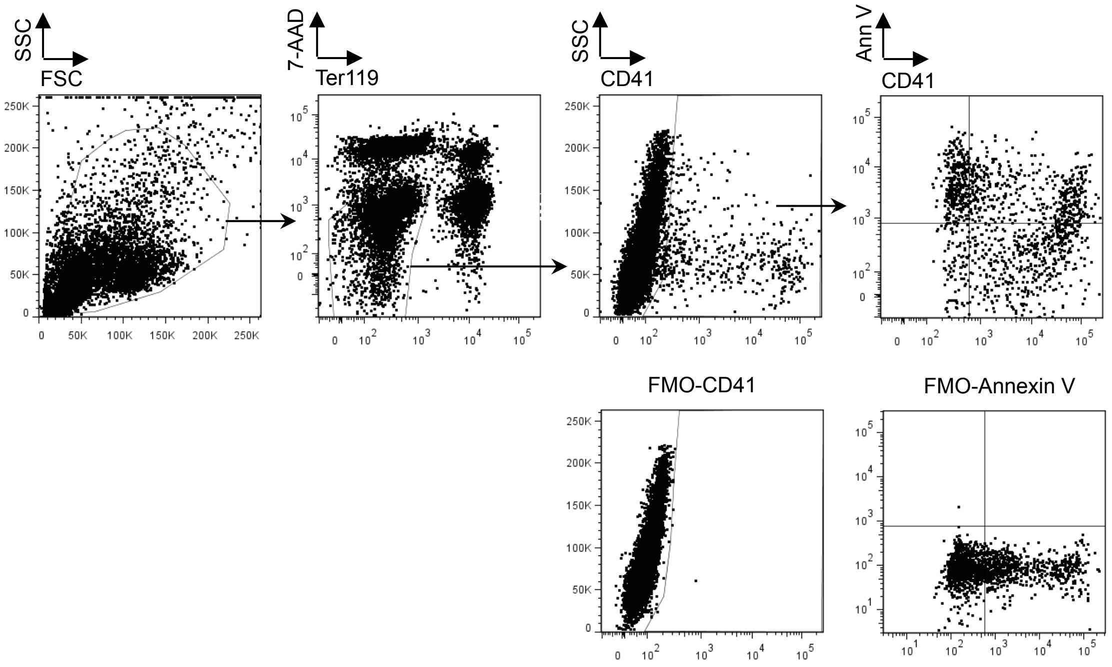

B

	Hematopoietic clusters and cells in Ao				CD45 in surrounding tissues*	
**Sample**	**Casp-3+ (total no.) clusters**	**Casp-3+ (total no.) cells**	**% apoptotic cells in intra-aortic clusters**	**% apoptotic intra-aortic clusters**	**CD45+Casp-3+ (total no.) cells**	**% apoptotic cells**
embryo 1	7 (16)	10 (71)	14.1	43.8	35 (79)	44.3
embryo 2	11 (26)	22 (106)	20.8	42.3	55 (117)	47.0
embryo 3	8 (16)	10 (78)	12.80	50.0	14 (45)	31.1
embryo 4	3 (12)	5 (48)	10.40	25.0	42 (91)	46.2
embryo 5	16 (29)	25 (119)	21.0	55.2	28 (78)	35.9

Fig. S2. Analysis of apoptotic cells in AGM region. (**A**) Gating strategy for analysis of Annexin V staining in the E10.5 CD41+ population. FMO controls for CD41 and Annexin V stainings are shown in the lower row. (**B**) Active Caspase3 staining in E11.5 intra-aortic clusters and CD45+ cells in the mesenchyme surrounding the dorsal aorta
